# Elevated expression of nuclear receptor-binding SET domain 3 promotes pancreatic cancer cell growth

**DOI:** 10.1038/s41419-021-04205-6

**Published:** 2021-10-06

**Authors:** Yihui Sun, Jiaming Xie, Shang Cai, Qian Wang, Zhenyu Feng, Yecheng Li, Jing-jing Lu, Wei Chen, Zhenyu Ye

**Affiliations:** 1 Department of General Surgery, The Second Affiliated Hospital of Soochow University, Suzhou, China; 2 Department of Radiotherapy and Oncology, The Second Affiliated Hospital of Soochow University, Suzhou, China; 3 Department of Radiotherapy and Oncology, Affiliated Kunshan Hospital of Jiangsu University, Kunshan, China

**Keywords:** Pancreatic cancer, Oncogenes

## Abstract

The nuclear receptor-binding SET domain 3 (NSD3) catalyzes methylation of histone H3 at lysine 36 (H3K36), and promotes malignant transformation and progression of human cancer. Its expression, potential functions and underlying mechanisms in pancreatic cancer are studied. Bioinformatics studies and results from local human tissues show that NSD3 is upregulated in human pancreatic cancer tissues, which is correlated with poor overall survival. In primary and established pancreatic cancer cells, NSD3 silencing (by shRNAs) or CRISPR/Cas9-induced NSD3 knockout potently inhibited cell proliferation, migration and invasion, while provoking cell cycle arrest and apoptosis. Conversely, ectopic expression of NSD3-T1232A mutation significantly accelerated proliferation, migration, and invasion of pancreatic cancer cells. H3K36 dimethylation, expression of NSD3-dependent genes (*Prkaa2*, *Myc*, *Irgm1*, *Adam12*, and *Notch3*), and mTOR activation (S6K1 phosphorylation) were largely inhibited by NSD3 silencing or knockout. In vivo, intratumoral injection of adeno-associated virus (AAV)-packed NSD3 shRNA potently inhibited pancreatic cancer xenograft growth in nude mice. These results suggest that elevated NSD3 could be an important driver for the malignant progression of pancreatic cancer.

## Introduction

Pancreatic cancer, mainly pancreatic ductal adenocarcinoma (PDAC), remains one of the most fatal malignancy in United States and around the world [1, 2]. Cancer statistical analyses have predicted that deaths from pancreatic cancer would be second after lung cancer by 2030 [1, 2]. Due to the lack of early diagnosis means and effective clinical treatments, the five-year overall survival of this devastating disease is still below 7% [1, 2]. Gemcitabine, approved by FDA at 1997, is still the standard treatment of pancreatic cancer [3, 4]. Genetic driving factors, including KRAS and several others, have been identified in pancreatic cancer [5, 6]. Unfortunately, targeted therapies for pancreatic cancer are far from satisfactory [7, 8]. Therefore, it is urgent to identify novel therapeutic targets and to explore effective treatments for pancreatic cancer [7, 8].

The nuclear receptor-binding SET domain (NSD) family has three primary members, including NSD1, NSD2, and NSD3 [9–12]. NSD3, also known as WHSC1L1, is a key SET domain-containing histone lysine methyltransferase [9–12]. NSD3 prefers to recognize and bind to the N-terminal peptides of histone H3 and can dimethylate or trimethylate histone H3 at lysine 36 (H3K36) [9–12]. In addition, NSD3 has two PWWP (the conserved sequence motif of Pro-Trp-Trp-Pro) domains and five PHD (plant homeodomain) fingers. These domains are important for chromatin-associated biological processes through interacting with the histones and DNA reader or modifier proteins [9–12]. Studies have shown that NSD3 is essential for regulating a number of key biological processes, including chromatin modification, transcriptional regulation, and DNA repair [9–12]. There are at least two major isoforms of NSD3, including the long full-length isoform containing the catalytic histone methyltransferase SET-domain and the short isoform which only has the first N-terminal PWWP domain [9–12].

NSD3 gene is located at chromosome 8p11.23, the locus with significant cancer relevance [13–17]. By catalyzing histone H3 methylation and interacting with different proteins, NSD3 has been proposed as an important oncogenic gene required for tumorigenesis and progression of human cancer [13–17]. Zhou et al., found that siRNA-induced silencing of NSD3 inhibited breast cancer cell invasion and cell cycle progression [16]. Irish et al., reported that NSD3 is amplified in aggressive luminal B-type breast cancer, important for ERα overexpression to drive cancer progression [18]. In osteosarcoma cells NSD3 silencing resulted in viability reduction, cell cycle arrest, and apoptosis activation [14]. Jones et al., reported that amplification of the NSD3-BRD4-CHD8 pathway was associated with poor overall survival and progression-free survival in ovarian cancers [15]. Recently, Jeong et al., found that NSD3-mediated H3K36 methylation is essential for NOTCH signaling activation to promote breast tumor initiation and metastatic progression [13]. Li et al., reported a novel NSD3 mutation (T1232A), the latter is catalytically hyperactive in promoting H3K36 methylation and cancer growth [10]. In the present study, we show that long full-length NSD3 is overexpressed in pancreatic cancer. It is required for pancreatic cancer cell growth in vitro and in vivo.

## Materials and methods

Antibodies for p70 S6 Kinase (S6K1, Thr389, #9205), S6K1 (#9202), NSD3 (WHSC1L1, full length, #92056), Cleaved Caspase Antibody Sampler Kit (#9929), and β-Tubulin (#2146) were purchased from Cell Signaling Technologies (Beverly, MA, USA). All sequences, primers, and viral constructs were produced by Shanghai Genechem Co. (Shanghai, China). Cell culturing reagents, including medium, FBS (fetal bovine serum) and antibiotics, were obtained from Hyclone (Shanghai, China). Cell Counting Kit -8 (CCK-8) was obtained from Dojindo (Kumamoto, Japan). Fluorescence dyes, including EdU (5-ethynyl-2′-deoxyuridine), TUNEL (terminal deoxynucleotidyl transferase dUTP nick-end labeling), and JC-1 (5,5′,6,6′-tetrachloro-1,1′,3,3′-tetraethyl-imidacarbocyanine) were provided by Thermo-Fisher Invitrogen (Shanghai, China).

### Cell culture

The established pancreatic cancer cell lines, including PANC-1, Bxpc-3 and MIA PaCa-2, were purchased from Shanghai Institute of Biological Science (Shanghai, China). Cells were maintained in RPMI plus 8% fetal bovine serum (FBS). Surgery-isolated fresh pancreatic cancer tissues from three PDAC patients, with written-informed consent (all male, 43/56/71 years old), were thoroughly washed in DMEM and 1 mM DTT (Sigma). Tissues were cut into small pieces and digested for 1 h. Single-cell suspensions were then pelleted and washed. Blood vessel cells, immune cells, and fibroblasts were carefully removed, and the remaining primary cancer cells were re-suspended in high glucose (25 mM) DMEM with 12% FBS, 2 mM glutamine, 1 mM pyruvate, 10 mM HEPES, 100 units/mL penicillin/streptomycin, 0.1 mg/mL gentamicin, and 2 g/L fungizone. Primary cancer cells from different patients were named as PanCa-1, PanCa-2, and PanCa-3. Same procedure was applied to normal human pancreatic tissues to establish primary pancreatic epithelial cells (“priEpi”). Cells were routinely subjected to mycoplasma and microbial contamination examination every 4–6 weeks. We also routinely checked population doubling time, colony forming efficiency, and morphology. All investigations using clinical human samples were in accordance with the principles expressed in the Declaration of Helsinki, with approval by the Ethics Committee of The Second Affiliated Hospital of Soochow University. All cells in this study are with wild-type (WT) *NSD3*.

### RNA extraction and quantitative real-time PCR (qRT-PCR)

Total RNA was isolated from the cells and tissues through using an EASYspin Plus Tissue/Cell RNA Extraction Kit (Aidlab Biotechnology, Beijing, China). RNA was reversely transcribed to cDNA via a ThermoScript First Strand cDNA Synthesis Kit (Aidlab Biotechnology). A SYBR Green PCR Kit (Applied Biosystems, Shanghai, China) was utilized for qRT-PCR assays under the ABI-7900 system. A ^ΔΔ^Ct method was employed to quantify targeted mRNA expression. *GAPDH* was always tested as the internal control and reference gene. The mRNA primers for *NSD3* (full length), *Prkaa2*, *Myc*, *Irgm1*, *Adam12*, *Notch3*, and *GAPDH* were described in previous studies [13, 19].

### Human tissues

A total of 12 primary pancreatic cancer (all PDAC) patients at The Second Affiliated Hospital of Soochow University were enrolled. The surgery-isolated pancreatic cancer tissues and matched surrounding normal pancreatic epithelial tissues were freshly obtained and mechanically dissociated. Tissues were lysed by the tissue lysis buffer (Biyuntian, Wuxi, China). Expressions of mRNAs and proteins in tissue lysates were tested by qRT-PCR and western blotting assays. The protocols of utilizing human specimens were approved by the Ethics Committee of The Second Affiliated Hospital of Soochow University, in accordance with the principles of Declaration of Helsinki, and written-informed consent was obtained from each participant.

### Western blotting

For each treatment, aliquots of 40 µg protein lysates (from tissue or cultured cells) were electro-transferred under the 10–12% SDS-PAGE gels, and were transferred to PVDF blots (Millipore, Shanghai, China). The blots were blocked (in 10% milk PBST solution) and were incubated with applied primary and secondary antibodies. Enhanced chemiluminescence (ECL) reagents (GE Healthcare, Shanghai, China) were utilized to detect antigen–antibody binding based on the molecular weight. The total gray of the targeted protein band was quantified via an ImageJ software (NIH, US). The untrimmed western blotting images were listed in Fig. S1.

### NSD3 shRNA

A set of five different shRNAs targeting full-length long *NSD3* were individually inserted into the GV369 lentiviral vector (Genechem). The construct was transfected to HEK-293T cells together with the lentivirus Helper plasmids (Genechem). The generated shRNA-expressing lentivirus were filtered and enriched. Pancreatic cancer cells were seeded onto a six-well plate at 50–60% confluence (in polybrene-containing complete medium), and shRNA-containing lentivirus were added. After 36 h, cells were then cultured in puromycin (2.5 μg/mL)-containing complete medium for 7–8 days. NSD3 silencing in the stable cells was verified by western blotting and qRT-PCR assays. The scramble non-sense lentiviral shRNA (“shC”, Genechem) was transduced to control pancreatic cancer cells. For in vivo studies, NSD3 shRNA sequence was inserted into a adeno-associated virus (AAV) construct (AAV9, Genechem. The construct was transfected to HEK-293 cells, generating NSD3 shRNA-expressing AAV. The virus was filtered and enriched.

### NSD3 expression

The mutant NSD3 (T1232A [10, 19], pY1174A) and the wild-type NSD3 cDNA were synthesized by Genepharm (Shanghai, China), which were sub-cloned into the GV369 vector (with Flag tag) [20]. The vector was then transfected into HEK-293 cells together with the lentivirus Helper plasmids (Genechem) using Lipofectamine 3000 (Invitrogen). At 2 days post-transfection, lentivirus was filtered, enriched, and added to primary human pancreatic cancer cells. Infections were allowed to proceed for 48 h. The lentiviral vector-expressing cells were selected post-infection in the presence of puromycin (2.5 μg/mL). Ectopic expression of NSD3-Flag or NSD3-T1232A-Flag in the stable cells was verified by western blot assays.

### NSD3 KO

On the first day of infection, a LentiCas9-puro construct (Genechem) was transduced to the primary human pancreatic cancer cells and cultured for 3 days. Puromycin was added to select stable cells. Cells were further transfected with a Lenti-NSD3-sgRNA (targeted DNA sequence, GGATACTGATTATATGAC) construct for 24 h, and the transfected cells were further distributed to 96-well plates. Cells were then subjected to NSD3 KO screening and single stable NSD3 KO pancreatic cancer cells were established.

### Cell viability

Cells with applied genetic modifications were plated into 96-well plates at 3 × 10^3^ cells per well and cultured for 96 h. Cell viability was examined using CCK-8 protocol (Dojindo). The absorbance of each well was measured at 450 nm.

### Colony formation

For the colony formation assays, pancreatic cancer cells with applied genetic modifications were initially seeded onto six-well plates (at 2 × 10^3^ cells per well). Cell culture medium was renewed every 2 days for a total of 10 days. Afterwards, large cell colonies were stained with crystal violet solution and counted manually.

### EdU staining

Briefly, pancreatic cancer cells with applied genetic modifications were seeded into 12-well plates (4 × 10^4^ cells per well) and cultured for 96 h. Cells were then stained with EdU. The nuclei were co-stained with DAPI and visualized under a laser scanning confocal microscope (Leica, Beijing, China). EdU-positive nuclei ratio (% vs. DAPI) from 2000 nuclei of five random microscope views (1 × 100 magnification) per treatment was calculated. The representative EdU/DAPI fluorescence images were presented as well.

### Transwell assays

The 8.0 µm pore “Transwell” inserts were utilized. Pancreatic cancer cells with applied genetic modification were added to the upper chambers (5 × 10^4^ cells per chamber in 1% FBS medium). Simultaneously, 400 µL of complete medium containing 12% FBS was placed in the lower chambers. The cells were allowed to migrate for 24 h. On the upper surface, the non-migrated cells were carefully removed using a wet cotton swab. The migrated cells on the lower surface were rinsed with PBS, fixed, and stained with crystal violet. For cell invasion assays, “Transwell” inserts were always coated with 25 µL of Matrigel (Sigma), and all other steps were the same.

### Cell cycle studies

Pancreatic cancer cells with applied genetic modifications were cultured for 72 h and fixed with 70% ethanol at 4 °C. Cells were then incubated with 500 µL of PBS containing 0.5 mg of RNase and stained with 25 µg/mL of propidium iodide (PI). Cells were then analyzed by FACS machine (BD Pharmingen).

### Caspase-3 activity

In brief, protein lysates (40 μg protein for each treatment) in caspase assay buffer were incubated with 7-amino-4-trifluoromethylcoumarin (AFC)-conjugated caspase-3 substrate. Reaction proceeded for 4 h, and the released AFC activity was examined by microplate reader with 505 nm emission.

### Single-stranded DNA (ssDNA) ELISA

Pancreatic cancer cells with the applied modifications were seeded into 12-well plates at 5 × 10^4^ cells/well. A ssDNA apoptosis ELISA Kit (Merck Millipore, Shanghai, China) was utilized to examine and quantify ssDNA contents, indicating DNA break intensity. The ELISA absorbance in each well was tested at 405 nm.

### TUNEL staining

Briefly, pancreatic cancer cells with applied genetic modifications were seeded into 12-well plates (4 × 10^4^ cells per well) and cultured for 96 h. Afterwards, cell nuclei were stained with TUNEL and DAPI. The nuclei were then washed and visualized under the confocal microscope (Leica). For each treatment, TUNEL-positive nuclei ratio (% vs. DAPI) from 2000 nuclei of five random microscope views (1 × 100 magnification) was calculated.

### Mitochondrial depolarization

In cells with mitochondrial depolarization JC-1 fluorescence dye will form monomers and emit green monomers [21]. Pancreatic cancer cells with applied genetic modifications were cultured for 72 h and stained with JC-1 (7.5 μg/mL). Cells were then washed and examined under a fluorescence spectrofluorometer (F-7000, Hitachi, Japan) at 490 nm. The representative JC-1 fluorescence images integrating both green and red (at 625 nm, normal mitochondrial membrane potential) fluorescence channels were presented.

### Xenograft assay

The nude mice, 4–5 weeks old, 18.5–19.0 g weight, half of them male, were purchased from the Experimental Animal Center of Medical School of Soochow University (Suzhou, China). Mice were housed under standard procedures. Pancreatic cancer cells were inoculated subcutaneously into the flanks of the mice. For each mouse, six million cells in 100 µL of saline/Matrigel, 1:1 v/v (no serum) were inoculated. Within 3 weeks, the volume of each tumor was approximately 100 mm^3^ (“Day-0”). Mice were then randomized into three groups. Ten mice per group were intratumorally injected with NSD3 shRNA AAV or the control shRNA AAV. AAV injection was performed daily for 7 days. Tumor parameters were recorded and tumor volumes were calculated using the following formula: *V* (volume) = 0.5328 × length × width× height (mm^3^) [22–24]. The animal procedures were approved by the Institute Animal Ethics Review Board of The Second Affiliated Hospital of Soochow University.

### Statistical analysis

The investigators were blinded to the group allocation for all in vitro experiments. Data were presented as mean ± standard deviation (SD). Statistics were analyzed by one-way ANOVA followed by a Scheffé and Tukey Test by SPSS 23.0 software (SPSS Inc., Chicago, IL, USA). The Student’s *t* test (Excel 2007) was applied to compare statistical difference between two groups. A *P*-value < 0.05 was considered to indicate statistical significance.

## Results

### NSD3 is overexpressed in pancreatic cancer tissues and cells

**Fig. 1:**
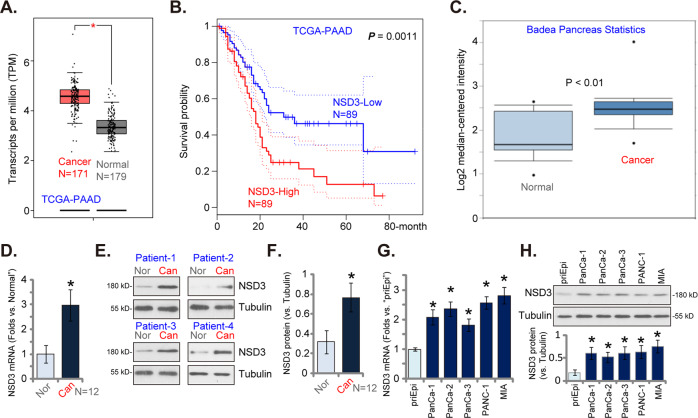
NSD3 is overexpressed in pancreatic cancer tissues and cells. TCGA-PAAD cohorts show relative *NSD3 mRNA* expression in 171 cases of pancreatic ductal adenocarcinoma (PDAC) tissues (“Cancer”) and 179 cases of normal pancreatic tissues (“Normal”) (**A**). Kaplan-Meier survival analyses of *NSD3*-low (*N* = 89) and *NSD3*-high (*N* = 89) PDAC patients (**B**). Badea Pancreas Statistics shows relative *NSD3* mRNA expression in PDAC cancer tissues (“Cancer”) and matched surrounding normal pancreatic tissues (“Normal”) (**C**). *NSD3* mRNA and protein expressions in pancreatic cancer tissues (“Can”) and matched surrounding normal pancreatic tissues (“Nor”) from 12 (*N* = 12) local PDAC patients were tested by qRT-PCR (**D**) and western blotting (**E**, **F**) assays. *NSD3* mRNA (**G**) and protein (**H**) expressions in described pancreatic cancer cells and epithelial cells (“priEpi”) were tested. Data were presented as mean ± standard deviation (SD). ^*^P < 0.05 vs. “Normal” tissues (**A**–**C**). ^*^P < 0.05 vs. “Nor” tissues (**D**, **F**). ^*^P < 0.05 vs. “priEpi” cells (**G** and **H**).

First The Cancer Genome Atlas (TCGA) PDAC cohort, annotated as PAAD-TCGA, was consulted to examine *NSD3* expression in human pancreatic cancer. Results show that *NSD3* mRNA expression in PDAC tissues (“Cancer”, *N* = 171) was significantly higher than that in the normal pancreatic tissues (“Normal”, *N* = 179) (Fig. 1A). Importantly, the average overall survival of patients with *NSD3*-high PDAC is worse than that in *NSD3*-low PDAC patients (*P* = 0.0011, Fig. 1B). Moreover, results retrieved from the Badea Pancreas Statistics also confirmed *NSD3* mRNA upregulation in PDAC patients (*P* < 0.05 vs. normal pancreatic tissues, Fig. 1C). These bioinformatics studies confirmed *NSD3* mRNA upregulation in human pancreatic cancer.

To further confirm the bioinformatics results, the fresh pancreatic cancer tissues (“Can”) and matched surrounding normal pancreatic tissues (“Nor”) from 12 (*N* = 12) local primary PDAC patients were obtained and NSD3 expression was tested. qRT-PCR assay results showed that *NSD3* mRNA in cancer tissues was significantly higher than that in normal tissues from local patients (Fig. 1D). Moreover, NSD3 protein (long form, same for all figures) upregulation in cancer tissues was detected in four representative PDAC patients (namely, “Patient-1 to Patient-4”) (Fig. 1E). All 12 sets of NSD3 blotting data were combined and results showed that NSD3 protein upregulation in pancreatic cancer tissues was significant (*P* < 0.05 vs. normal pancreatic tissues, Fig. 1F).

We also tested NSD3 expression in pancreatic cancer cells. Primary human pancreatic cancer cells derived from three individual patients, PanCa-1/-2/-3, as well as the established cell lines, PANC-1 and MIA PaCa-2 (“MIA”), were tested. As shown, in the primary and established pancreatic cancer cells, *NSD3* mRNA (Fig. 1G) and protein (Fig. 1H) expressions are significantly higher than those in the primary pancreatic epithelial cells (“priEpi”) (Fig. 1G, H). These results together show that NSD3 is overexpressed in both human pancreatic cancer tissues and cells.

### NSD3 shRNA inhibits pancreatic cancer cell survival, proliferation and migration

**Fig. 2:**
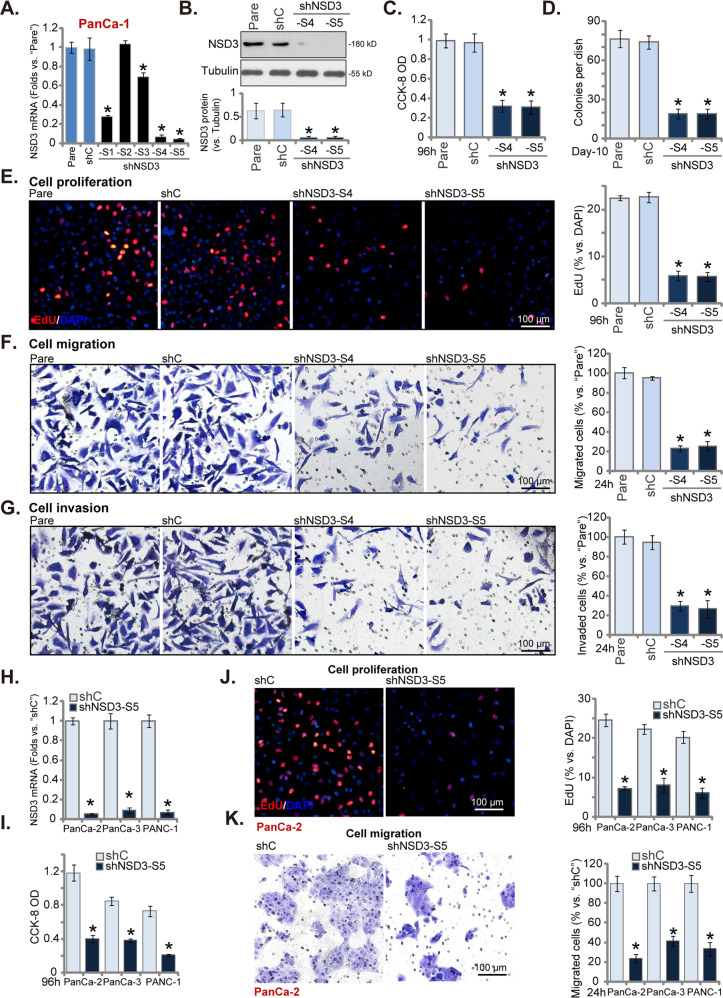
NSD3 shRNA inhibits pancreatic cancer cell survival, proliferation and migration. The primary human pancreatic cancer cells (PanCa-1/-2/-3, derived from three different patients) or PANC-1 cells, stably expressing applied NSD3 shRNA or control shRNA (“shC”), were established. Expressions of *NSD3* mRNA and listed proteins were examined by qRT-PCR (**A**, **H**) and western blotting (**B**) assays. Cells were further cultured for applied time periods, cell viability (CCK-8 assays, **C**, **I**), colony formation (**D**) and proliferation (EdU staining assays, **E**, **J**), as well as cell migration (“Transwell” assays, **F**, **K**) and invasion (“Matrigel Transwell” assays, **G**) were tested. “Pare” stands for the parental control cells. Data were presented as mean ± standard deviation (SD, *n* = 5). ^*^P < 0.05 vs. “shC” cells. Experiments in this figure were repeated five times with similar results obtained. Scale bar = 100 μm (**E**–**G**, **J**, **K**).

The shRNA strategy was applied to silence NSD3. As described, a set of five different shRNAs, targeting non-overlapping sequences of NSD3 (“shNSD3-S1/2/3/4/5”), were individually transduced to primary human pancreatic cancer cells (“PanCa-1”). Stable cells were established via puromycin selection. *NSD3* mRNA expression in the stable cells was examined by qRT-PCR assays. Four out of five applied NSD3 shRNAs resulted in significant *NSD3* mRNA downregulation (Fig. 2A). Among them, shNSD3-S4 and shNSD3-S5 resulted in most significant *NSD3* mRNA silencing (Fig. 2A). The two shRNAs were utilized for further experiments. Western blotting assay results, Fig. 2B, demonstrated that NSD3 protein levels were dramatically decreased in stable PanCa-1 cells expressing shNSD3-S4 or shNSD3-S5. CCK-8 assays were performed to test cell viability. We showed that shRNA-induced silencing of NSD3 significantly inhibited viability (CCK-8 OD) of PanCa-1 cells (Fig. 2C). In addition, the number of viable PanCa-1 cell colonies was dramatically decreased after NSD3 silencing (Fig. 2D).

Evidenced by decreased EdU-positive nuclei ratio, we showed that cell proliferation was inhibited in PanCa-1 cells with shNSD3-S4 or shNSD3-S5 (Fig. 2E). “Transwell” and “Matrigel Transwell” assays were carried out to test cell migration and invasion, respectively. Results showed that shRNA-induced silencing of NSD3 potently inhibited PanCa-1 cell migration (Fig. 2F) and invasion (Fig. 2G). Therefore, NSD3 silencing by targeted shRNAs significantly inhibited PanCa-1 cell viability, proliferation, migration and invasion. Unsurprisingly, the scramble control shRNA (“shC”) failed to significantly alter NSD3 expression (Fig. 2A, B) and PanCa-1 cell functions (Fig. 2C–G).

Whether NSD3 silencing exerted similar functions in other pancreatic cancer cells was also tested. Primary human pancreatic cancer cells, derived from two other patients (PanCa-2 and PanCa-3), as well as established cell lines (PANC-1, MIA PaCa-2, and Bxpc-3), were tested. These cells were infected with shNSD3-S5 lentivirus, and stable cells established after puromycin selection. shNSD3-S5 resulted in over 85–90% reduction of *NSD3* mRNA in the primary and established pancreatic cancer cells (Figs. 2H and S2A). Importantly, shNSD3-S5 largely inhibited cell viability (CCK-8 OD, Fig. 2I), proliferation (tested by EdU-positive nuclei ratio, Figs. 2J and S2B) and migration (“Transwell” assays, Figs. 2K and S2C) in the pancreatic cancer cells. These results showed that NSD3 silencing exerted significant tumor-suppressive functions in cultured pancreatic cancer cells.

### NSD3 shRNA induces cell cycle arrest and apoptosis activation in pancreatic cancer cells

**Fig. 3:**
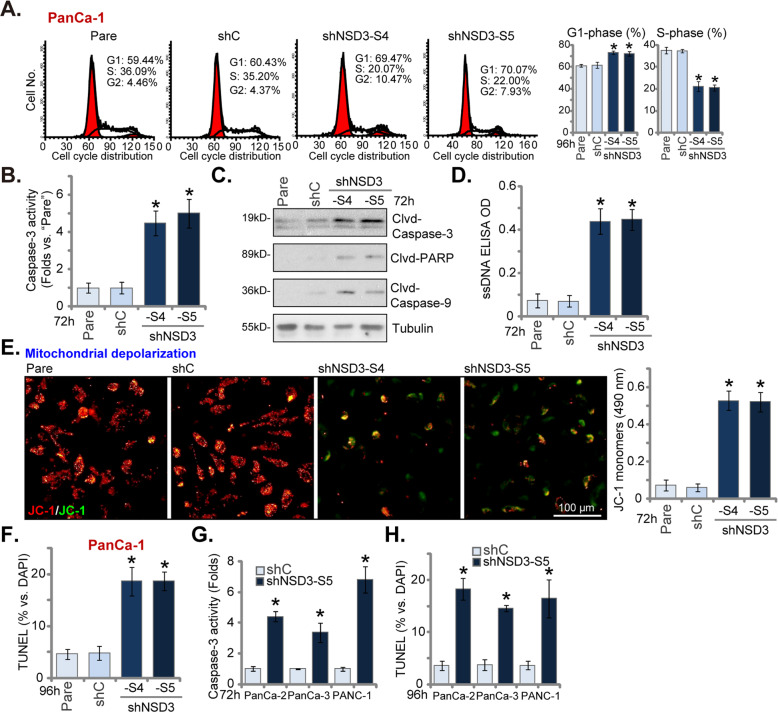
NSD3 shRNA induces cell cycle arrest and apoptosis activation in pancreatic cancer cells. The primary human pancreatic cancer cells (PanCa-1/-2/-3, derived from different patients) or PANC-1 cells, stably expressing applied NSD3 shRNA or scramble control shRNA (“shC”), were established and cultured for applied time periods. Cell cycle distribution was detected by PI-FACS assays (**A**); caspase-3 activity (**B**, **G**), expression of apoptosis-related proteins (**C**) and single stand DNA (ssDNA) contents (ELISA assays, **D**) were tested; JC-1 dye assays were performed to examine mitochondrial depolarization, and JC-1 green monomer fluorescence intensity was quantified (**E**); cell apoptosis was tested by nuclear TUNEL staining (**F**, **H**). “Pare” stands for the parental control cells. Data were presented as mean ± standard deviation (SD, *n* = 5). ^*^P < 0.05 vs. “shC” cells. Experiments in this figure were repeated five times with similar results obtained. Scale bar = 100 μm (**E**).

In pancreatic cancer cells, proliferation inhibition could possibly be due to cell cycle disruption. Since NSD3 shRNA inhibited pancreatic cancer cell proliferation, we therefore analyzed its effect on cell cycle progression. PI-FACS assays were performed and results showed that G1 phase percentage was significantly increased in stable PanCa-1 cells expressing shNSD3-S4 or shNSD3-S5 (Fig. 3A), while S-phase percentage was decreased (Fig. 3A). These results implied that NSD3 shRNA led to G1-S arrest in PanCa-1 cells. When testing cell apoptosis, we showed that the caspase-3 activity was significantly increased in NSD3 shRNA-bearing PanCa-1 cells (Fig. 3B). Furthermore, levels of cleaved caspase-3, caspase-9, and PARP [poly (ADP-ribose) polymerase] were significantly increased (Fig. 3C). In addition, ssDNA (reflected by ELISA OD) was accumulated in NSD3-silenced PanCa-1 cells, indicating increased DNA damage (Fig. 3D). With NSD3 silencing in PanCa-1 cells, significant mitochondrial depolarization, evidenced by JC-1 green monomers accumulation, was detected (Fig. 3E).

Importantly, NSD3 silencing induced apoptosis activation in PanCa-1 cells, as the TUNEL-positive nuclei ratio was significantly increased in stable PanCa-1 cells with shNSD3-S4 or shNSD3-S5 (Fig. 3F). In established PANC-1 cells and other primary human pancreatic cancer cells (PanCa-2 and PanCa-3), NSD3 silencing by shNSD3-S5 (see Fig. 2) significantly increased caspase-3 activity (Fig. 3G) and TUNEL-positive nuclei ratio (Fig. 3H), indicating apoptosis activation. NSD3 silencing by shNSD3-S5 induced apoptosis activation in MIA PaCa-2 and Bxpc-3 cells, evidenced by TUNEL-positive nuclei ratio increase (Fig. S2D). These results showed that NSD3 silencing induced cell cycle arrest and apoptosis activation in pancreatic cancer cells.

### NSD3 KO by CRISPR/Cas9 exerts tumor-suppressive functions in pancreatic cancer cells

**Fig. 4:**
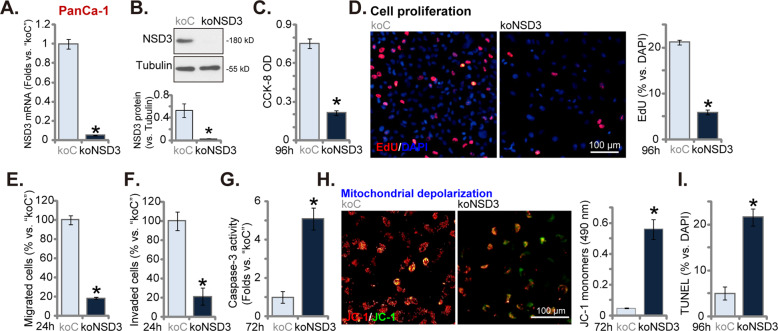
NSD3 KO by CRISPR/Cas9 exerts tumor-suppressive functions in pancreatic cancer cells. Single stable PanCa-1 primary pancreatic cancer cells, bearing the lenti-CRISPR/Cas9 construct encoding NSD3 sgRNA (“koNSD3”) or the lenti-CRISPR/Cas9 empty vector (“koC”), were established; expression of *NSD3* mRNA and protein was examined by qRT-PCR (**A**) and western blotting (**B**) assays, respectively. Cells were further cultured for applied time periods, cell viability (CCK-8 assays, **C**), proliferation (nuclear EdU staining assays, **D**), as well as cell migration (“Transwell” assays, **E**) and invasion (“Matrigel Transwell” assays, **F**) were tested, with results quantified); caspase-3 activity (**G**), mitochondrial depolarization (JC-1 staining assays, **H**) and cell apoptosis (TUNEL staining assays, **I**) were tested as well. Data were presented as mean ± standard deviation (SD, *n* = 5). ^*^P < 0.05 vs. “koC” cells. Experiments in this figure were repeated five times with similar results obtained. Scale bar = 100 μm (**D**, **H**).

To further support the function of NSD3 in pancreatic cancer cells, CRISPR/Cas9 strategy was applied to complete knockout NSD3. As described, a lenti-CRISPR/Cas9 construct encoding NSD3 sgRNA was transduced to PanCa-1 cells. Single stable cells, namely koNSD3 cells, were established after screening. As demonstrated, *NSD3* mRNA (Fig. 4A) and protein (Fig. 4B) expressions were completely depleted in the koNSD3 PanCa-1 cells. When compared to control cells with the lenti-CRISPR/Cas9 empty vector (“koC”), cell viability (CCK-8 OD) was significantly decreased in koNSD3 cells (Fig. 4C). Furthermore, CRISPR/Cas9-induced NSD3 KO largely inhibited PanCa-1 cell proliferation (testing by EdU-positive nuclei ratio, Fig. 4D), migration (Fig. 4E), and invasion (Fig. 4F). In contrast, the caspase-3 activity was significantly increased in koNSD3 PanCa-1 cells (Fig. 4G). JC-1 green monomer accumulation was detected in koNSD3 cells, indicating mitochondrial depolarization (Fig. 4H). Moreover, NSD3 KO induced apoptosis activation in PanCa-1 cells, and the TUNEL-positive nuclei ratio was significantly increased (Fig. 4I).

Similarly in MIA PaCa-2 cells and Bxpc-3 cells, stable transfection of the NSD3 sgRNA lenti-CRISPR/Cas9 construct caused *NSD3* mRNA depletion (“koNSD3”, Fig. S2A). When compared to the control cells with koC, NSD3 KO potently inhibited cell proliferation (EdU-positive nuclei ratio reduction, Fig. S2B) and migration (“Transwell” assays, Fig. S2C), while provoking apoptosis activation (TUNEL-positive nuclei ratio increase, Fig. S2D) in the established pancreatic cancer cells. These results showed that CRISPR/Cas9-induced NSD3 KO produced significant anti-tumor activity in cultured pancreatic cancer cells.

### NSD3-T1232A accelerates pancreatic cancer cell proliferation, migration, and invasion

Recent studies have shown that the cancer-associated NSD3 mutation, T1232A, presented with increased catalytic activity for H3K36 dimethylation (H3K36me2) [10, 19]. Ectopic expression of NSD3-T1232A could robustly promote tumorigenesis [10, 19]. We therefore tested the potential role of NSD3-T1232A on pancreatic cancer cell behaviors. The GV369 construct encoding the wild-type (WT) NSD3-Flag or the mutant NSD3-T1232A-Flag was separately transduced to koNSD3 PanCa-1 primary cells. After selection by puromycin, stable cells were established. Control koNSD3 PanCa-1 cells were with the GV369 empty vector (“Vec”).

**Fig. 5:**
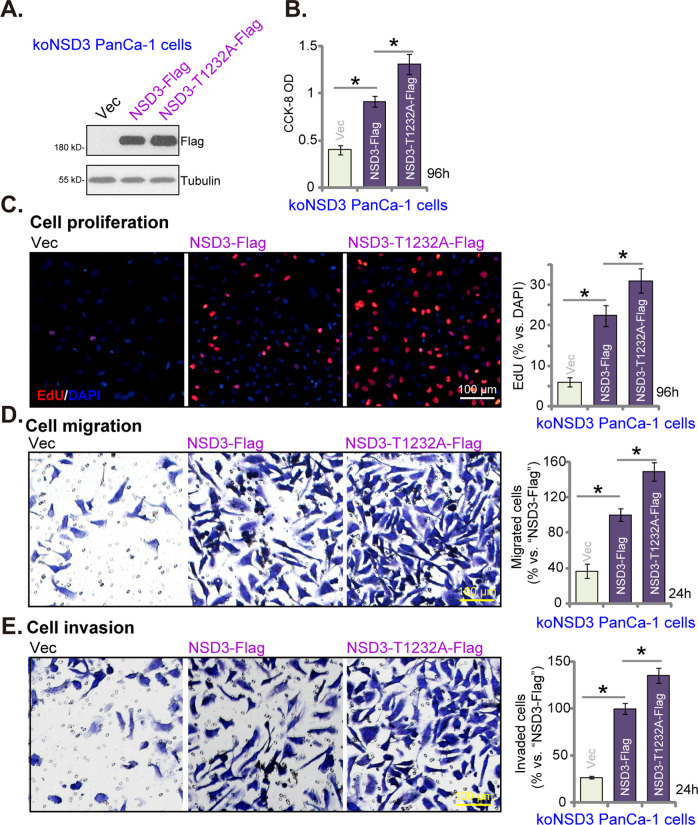
NSD3-T1232A accelerates pancreatic cancer cell proliferation, migration and invasion. Single stable PanCa-1 primary pancreatic cancer cells bearing the lenti-CRISPR/Cas9 construct encoding NSD3 sgRNA (“koNSD3”) were further transduced with NSD3-T1232A-Flag construct, the wild-type NSD3-Flag construct or empty vector (“Vec”); Expression of listed proteins was examined by western blotting assays (**A**). Cells were further cultured for applied time periods, cell viability (CCK-8 assays, **B**), proliferation (EdU staining assays, **C**), cell migration (“Transwell” assays, **D**), and invasion (“Matrigel Transwell” assays, **E**) were tested. Data were presented as mean ± standard deviation (SD, *n* = 5). ^*^P < 0.05. Experiments in this figure were repeated five times with similar results obtained. Scale bar = 100 μm (**C**–**E**).

Western blotting assay results (Fig. 5A) confirmed the ectopic expression of WT NSD3-Flag and mutant NSD3-T1232A-Flag in koNSD3 PanCa-1 cells. Functional studies demonstrated that ectopic expression of WT NSD3 restored cell viability (CCK-8 OD, Fig. 5B), proliferation (Fig. 5C), migration (Fig. 5D), and invasion (Fig. 5E) in the koNSD3 cells. Importantly, as compared to WT NSD3, expression of NSD3-T1232A was more potent in augmenting cell viability (Fig. 5B), proliferation (Fig. 5C), migration (Fig. 5D), and invasion (Fig. 5E) in koNSD3 cancer cells. These results implied that NSD3-T1232A accelerated pancreatic cancer cell proliferation, migration, and invasion.

### NSD3 is essential for H3K36 methylation, oncogenic genes expression and mTOR activation in pancreatic cancer cells

**Fig. 6:**
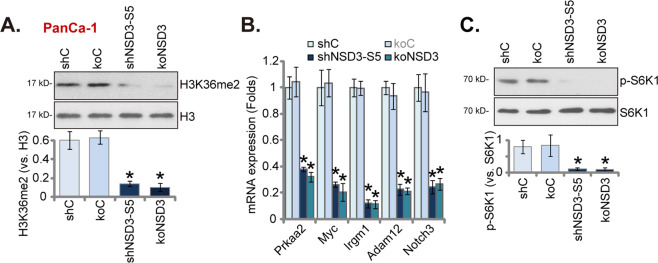
NSD3 is essential for H3K36 methylation, oncogenic genes expression and mTOR activation in pancreatic cancer cells. Stable PanCa-1 cells, bearing shNSD3-S5, the scramble control shRNA (“shC”), the lenti-CRISPR/Cas9 construct encoding NSD3 sgRNA (“koNSD3”) or the lenti-CRISPR/Cas9 empty vector (“koC”), were established and cultured; expression of listed proteins was tested by western blotting assays (**A**, **C**); listed mRNAs were examined by qRT-PCR assays (**B**). Data were presented as mean ± standard deviation (SD, *n* = 5). ^*^P < 0.05 vs. “shC” cells. Experiments in this figure were repeated five times with similar results obtained.

Studies have shown that NSD3 catalyzes H3K36 methylation to transcriptionally activate multiple oncogenic genes, thereby promoting cancer progression [10, 13–17, 19]. In PanCa-1 pancreatic cancer cells, NSD3 silencing (by shNSD3-S5, see Figs. 2 and 3) or KO (koNSD3, see Fig. 4) largely inhibited H3K36 dimethylation (H3K36me2) (Fig. 6A). NSD3-dependent genes, including *Prkaa2*, *Myc*, *Irgm1*, *Adam12*, and *Notch3*, were significantly downregulated in PanCa-1 cells with NSD3 silencing or KO (Fig. 6B). H3K36m2-dependent genes were also reported to be required for mTOR cascade activation [10, 19]. We found that S6K1 phosphorylation, the indicator of mTOR activation [25, 26], was inhibited as well after NSD3 silencing or KO (Fig. 6C).

### NSD3 shRNA injection inhibits PanCa-1 xenograft tumor growth in nude mice

**Fig. 7:**
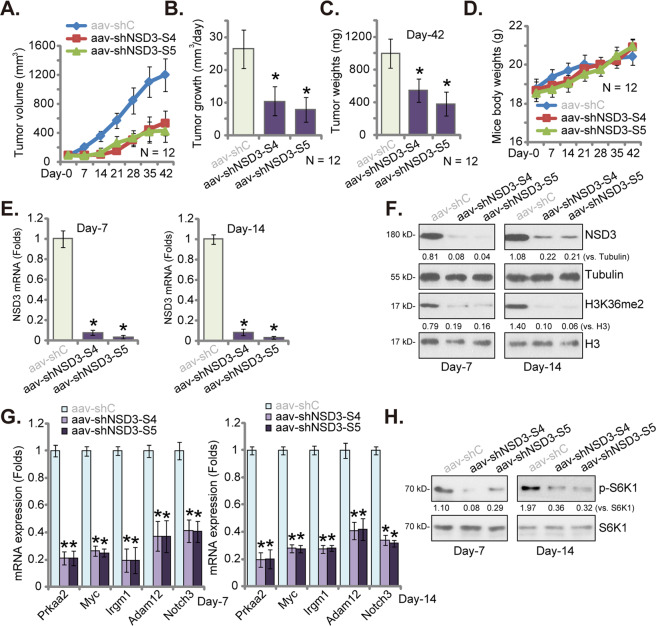
NSD3 shRNA injection inhibits PanCa-1 xenograft tumor growth in nude mice. The PanCa-1 xenograft-bearing nude mice were subjected to intratumoral injection of AAV-packed shNSD3-S4 (“aav-shNSD3-S4”), AAV-packed-shNSD3-S5 (“aav-shNSD3-S5”) or AAV-packed shC (“aav-shC”), daily for 7 consecutive days; tumor volumes (**A**) and mice body weights (**D**) were recorded every 7 days for a total of 42 days. The estimated daily tumor growth, in mm^3^ per day, was calculated using the described formula (**B**). All tumors were carefully isolated at Day-42 and weighted individually (**C**). At Day-7 and Day-14, one tumor of each group was carefully separated and total six tumors were acquired. Expression of listed mRNAs and proteins in tumor tissue lysates were tested by qRT-PCR (**E**, **G**) and western blotting (**F**, **H**) assays, respectively. Targeted proteins were quantified and normalized to the corresponding loading controls (**F**, **H**). Data were presented as mean ± standard deviation (SD). *N* = 12 stands for 12 mice per group (**A**–**D**). ^*^P < 0.05 vs. “aav-shC” group.

At last, we tested the potential function of NSD3 on pancreatic cancer cell growth in vivo. PanCa-1 cells (6 × 10 ^6^ cells per mouse) were s.c. injected to the right flanks of nude mice and xenograft tumors were established within 3 weeks. Mice were then randomly assigned into three groups, receiving intratumoral injection of AAV-packed shNSD3-S4 (“aav-shNSD3-S4”), AAV-packed-shNSD3-S5 (“aav-shNSD3-S5”), or AAV-packed shC (“aav-shC”). AAV injection was performed daily for 7 consecutive days. Tumor growth curve results, Fig. 7A, demonstrated that the growth of shNSD3 AAV-injected tumors was significantly slower than tumors with shC AAV injection. Volumes of tumors bearing NSD3 shRNA were significantly lower than shC-bearing tumors (Fig. 7A). The following formula was utilized to calculate the estimated daily tumor growth: [Tumor volume (in mm^3^) at Day-42 subtracting tumor volume (in mm^3^) at Day-0]/42. Results again showed that NSD3 shRNA AAV injection potently inhibited PanCa-1 xenograft growth in nude mice (Fig. 7B). At Day-42 all tumors were isolated from the mice and weighted individually. Results showed that shNSD3 AAV-injected tumors were significantly lighter than shC AAV-injected tumors (Fig. 7C). Therefore, NSD3 shRNA AAV injection inhibited PanCa-1 cell growth in vivo. As expected, the mice body weights were not significantly different between the three groups (Fig. 7D).

At Day-7 and Day-14, one tumor of each group was carefully separated and a total of six tumors were acquired. The fresh tumors were cut into small pieces and dissolved in tissue lysis buffer. The qRT-PCR assay results, Fig. 7E, confirmed that *NSD3* mRNA levels were robustly decreased in shNSD3 AAV-injected tumor tissues. Western blotting assays results showed that NSD3 protein levels and H3K36me2 were decreased as well (Fig. 7F). In addition, mRNA levels of NSD3-dependent genes (*Prkaa2*, *Myc*, *Irgm1*, *Adam12*, and *Notch3*) were significantly downregulated in NSD3 shRNA-injected xenograft tissues (Fig. 7G). S6K1 phosphorylation reduction, indicating mTOR inactivation, was detected as well in NSD3 shRNA-injected xenograft tissues (Fig. 7H). Therefore, these results implied that H3K36 methylation, oncogenes expression and mTOR activation were inhibited in PanCa-1 xenograft tissues with NSD3 silencing. The findings are therefore consistent with in vitro results (see Fig. 6).

## Discussion

NSD3 is a principal 8p11-12 amplicon-associated oncogenic driver, whose overexpression and/or mutations are associated with malignant transformation and progression of multiple human cancers [10, 19]. In the present study, the bioinformatics studies show that *NSD3* is overexpressed in pancreatic cancers, correlating with poor overall survival. In addition, our results found that *NSD3* mRNA and protein levels in local pancreatic cancer tissues were significantly higher than those in matched surrounding pancreatic tissues. In addition, NSD3 overexpression was also detected in different human pancreatic cancer cells. Therefore, aberrant NSD3 overexpression in pancreatic cancer implied a potential role of this gene in cancer carcinogenesis and progression.

Indeed, in both established and primary human pancreatic cancer cells, shRNA-induced silencing of NSD3 induced significant anti-tumor activity by inhibiting cell viability, proliferation, migration, and invasion. Cell cycle arrest and apoptosis were detected in NSD3-silenced pancreatic cancer cells. Moreover, CRISPR/Cas9-induced complete NSD3 KO inhibited pancreatic cancer cell growth and induced significant apoptosis activation. Conversely, ectopic expression of the mutant and constitutively active NSD3 (T1232A) accelerated pancreatic cancer cell proliferation, migration, and invasion. In vivo, NSD3 shRNA AAV injection potently inhibited PanCa-1 xenograft tumor growth in nude mice. These results implied that NSD3 could be a novel oncogenic gene and therapeutic target for pancreatic cancer.

Recent studies have discovered that NSD3 that contains mutation at T1232A is catalytically hyperactive for H3K36me2. In head and neck squamous cell carcinoma (HNSCC) cells and lung cancer cells, ectopic expression of NSD3-T1232A promoted cancer cell proliferation in vitro and xenograft tumor growth in vivo [10, 19]. In addition, NSD3-T1232A expression significantly decreased overall survival in mouse models of lung cancer [19]. In line with these findings, we found that ectopic expression of NSD3-T1232A significantly accelerated proliferation, migration, and invasion of pancreatic cancer cells. Further studies will be needed to explore the function and underlying mechanisms of this mutation (and possible other NSD3 mutations) in the progression of pancreatic cancer.

Jeong et al., recently reported that NSD3-catalyzed H3K36me2 is crucial for breast cancer initiation, metastasis, and malignant progression [13]. NSD3 directly interacted with EZH2 and RNA polymerase II to stimulate H3K36 methylation-dependent transcriptional activation of NOTCH receptor cleavage-associated genes (including *Adam12*, *DLL4,* and *Notch3*), thereby activating Notch signaling to promote breast tumor progression [13]. Yuan et al., have recently found that NSD3-T1232A could drive squamous cell lung cancer progression by robustly increasing H3K36m2 and transcriptional activation of several key oncogenic genes, including *Prkaa2*, *Myc*, and *Irgm1* [19]. Here we showed that NSD3 silencing (by targeted shRNA) or KO (using CRISPR/Cas9 method) in pancreatic cancer cells potently inhibited H3K36m2 and expression of these oncogenic genes (*Prkaa2*, *Myc*, *Irgm1*, *Adam12,* and *Notch3*). In vivo, H3K36me2 and NSD3-dependent genes were significantly decreased in PanCa-1 xenograft tumor tissues with NSD3 shRNA AAV injection. Studies have shown that NSD3-dependent genes (*Prkaa2*, *Myc*, *Irgm1*, *Adam12*, and *Notch3*) could exert different cancer-promoting functions in pancreatic cancer [27–31]. These results implied that NSD3-catalyzed H3K36 methylation induced transcriptional activation of multiple oncogenic genes, thereby promoting malignant progression of pancreatic cancer.

mTOR pathway is vital for modulating key cancer cell behaviors, including cell growth, proliferation, migration and survival, as well as apoptosis resistance, metabolism, and angiogenesis [26, 32–34]. Hyperactivation of this cascade, due to various gene mutations, is of most frequent occurrence in pancreatic cancer [35, 36]. It is therefore is a key therapeutic target [37–41]. A recent study has demonstrated that NSD3-catalyzed H3K36 methylation stimulated transcription activation of key genes required for mTOR activation [19]. Here we found that mTOR activation (S6K1 phosphorylation) in pancreatic cancer cells was largely inhibited by NSD3 shRNA or KO. Furthermore, S6K1 phosphorylation was inhibited in pancreatic cancer xenograft tumor tissues with NSD3 shRNA AAV injection. Therefore, regulating mTOR activation could be another key mechanism responsible for NSD3-driven pancreatic cancer progression. The underlying mechanisms may warrant further characterizations.

## Conclusion

Accumulation of various inherited or acquired gene mutations are essential for the tumorigenesis and progression of pancreatic cancer [42–46]. Activating mutation of *KRAS* as well as the inactivation of several key tumor suppressor genes, including *CDKN2A*, *p16INK4A*, *TP53*, and *SMAD4*, are prevalent in the different stages of pancreatic cancers and also in precursor lesions [42–46]. Yet, with the advances in the understanding of pancreatic cancer genetics, the prognosis of the devastating disease is still quite poor [1, 47]. Thus, the pathogenesis needs to be further clarified to improve pancreatic cancer therapeutics [42–46]. Here we found that NSD3 catalyzes H3K36 methylation and functions a potential driver for the malignant progression of pancreatic cancer. Targeting NSD3 could be a novel strategy to treat this devastating disease. Further studies, however, are needed to explore the relationship between NSD3 and these known mutated genes in pancreatic cancers.

## Supplementary information

Figure S1.

Figure S2.
