# Supplementary figures and images for: Elevated expression of nuclear receptor-binding SET domain 3 promotes pancreatic cancer cell growth
Source: Cell Death Dis. 2021 Oct 6;12(10):913. doi: 10.1038/s41419-021-04205-6 (PMC8494902; doi:10.1038/s41419-021-04205-6)

Figure S1.

Figure 1.

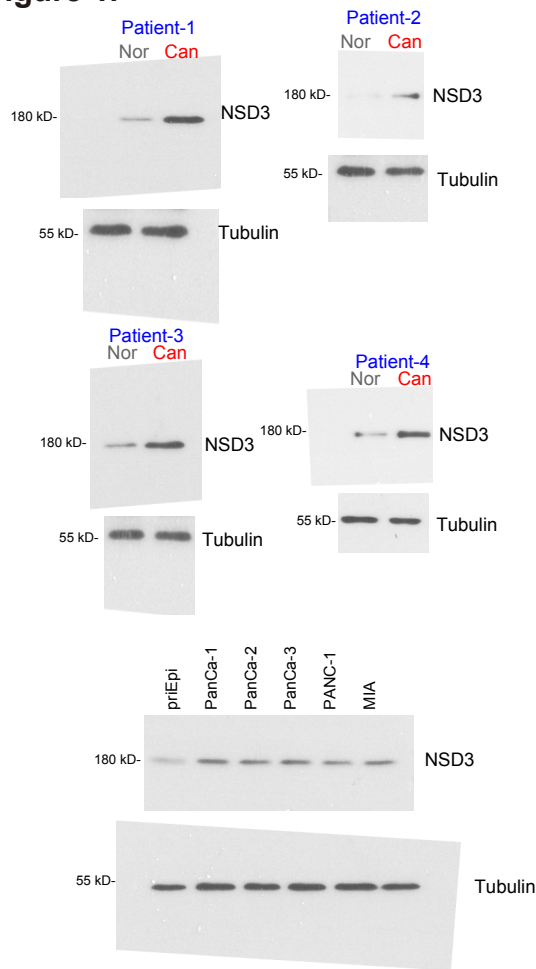

Figure 2.

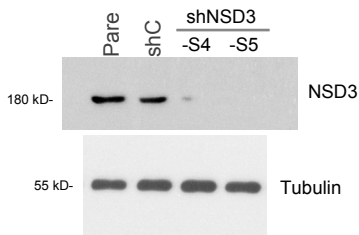

Figure 4.

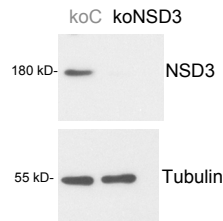

Figure 3.

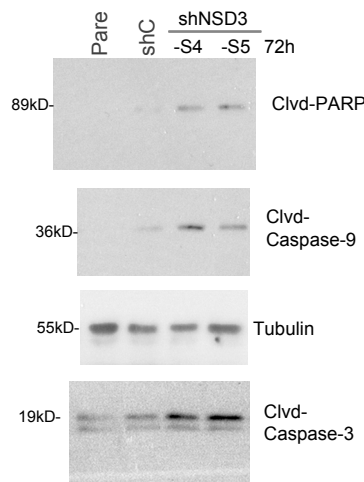

Figure 5

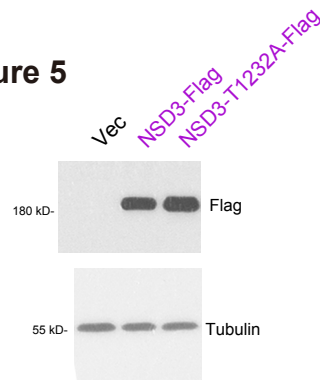

Figure 6.

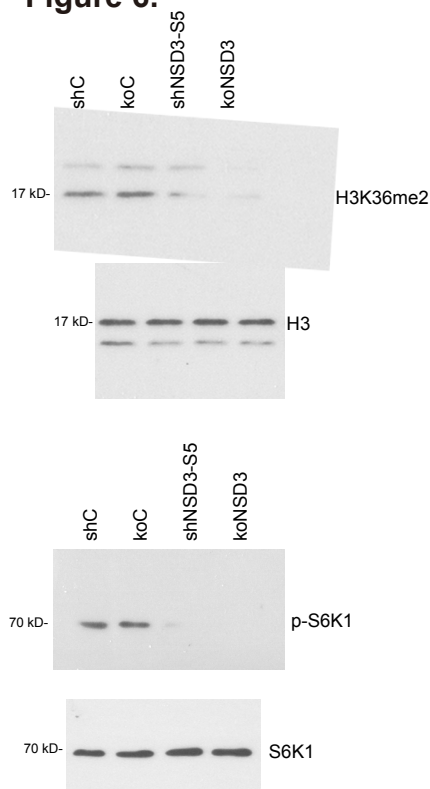

Figure 7.

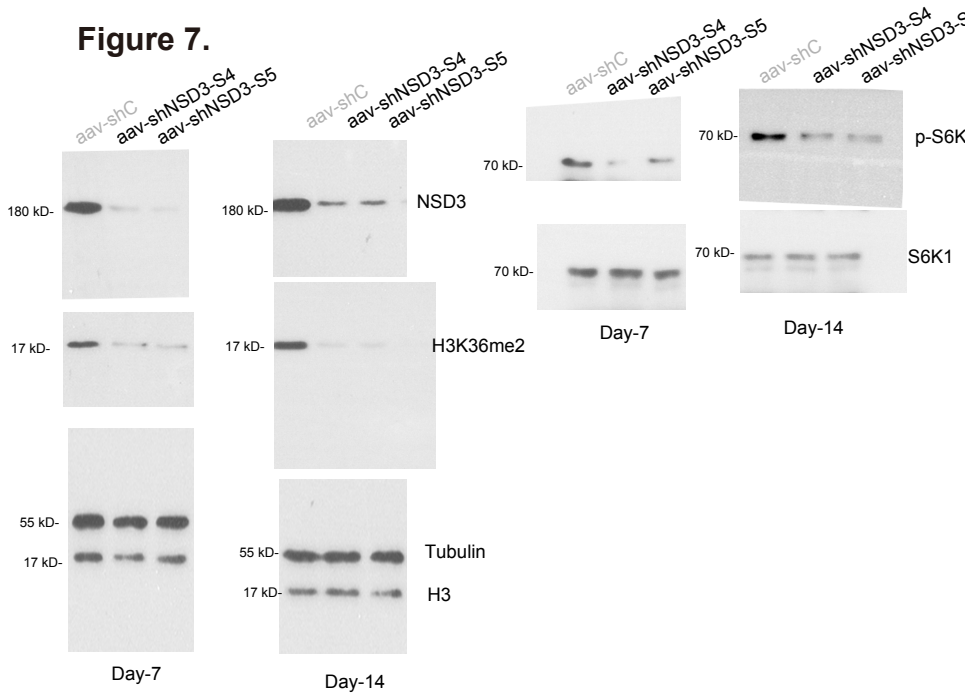

Supplement: Supplementary file 1 — Figure S1. [file 41419_2021_4205_MOESM1_ESM.pdf]
