# Supplementary material for: Elevated expression of nuclear receptor-binding SET domain 3 promotes pancreatic cancer cell growth
Source: Cell Death Dis. 2021 Oct 6;12(10):913. doi: 10.1038/s41419-021-04205-6 (PMC8494902; doi:10.1038/s41419-021-04205-6)
Supplement: Supplementary file 2 — Figure S2. [file 41419_2021_4205_MOESM2_ESM.pdf]

Figure S2

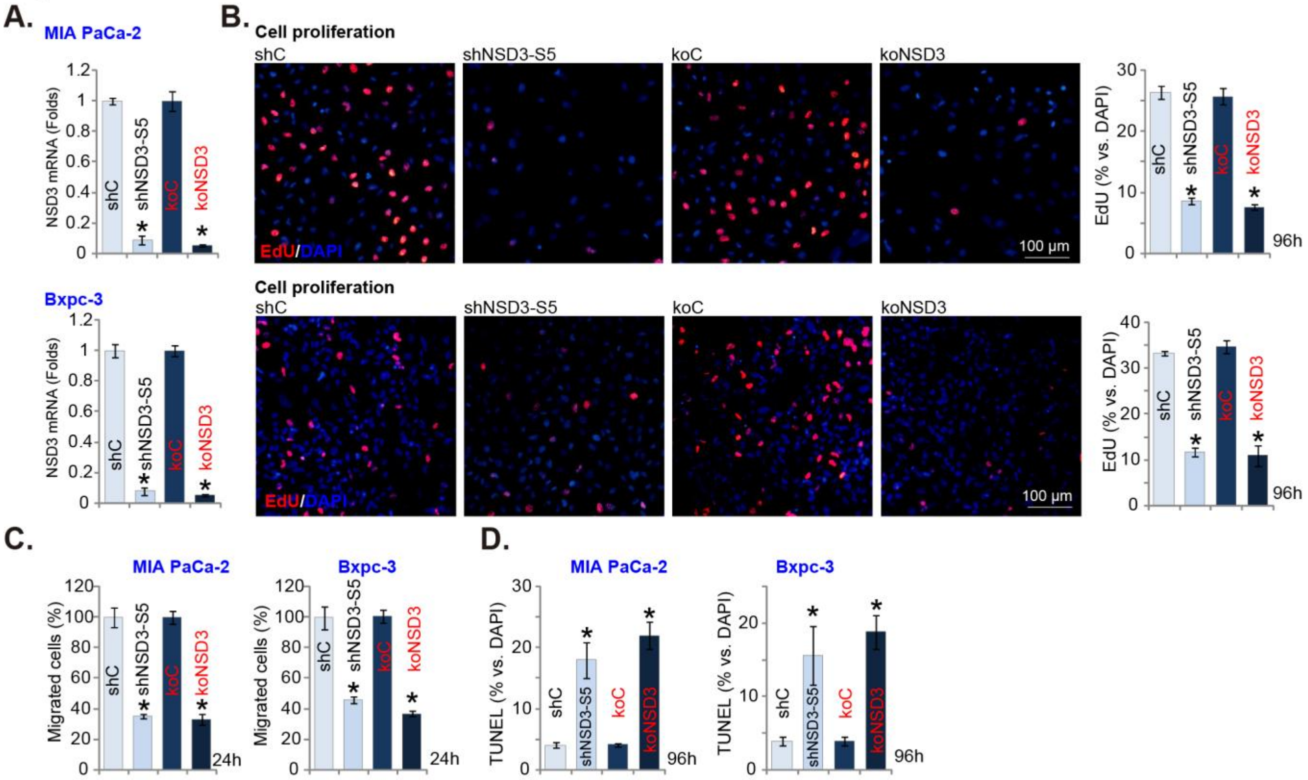

Figure S2. PaCa-2 cells and Bxpc-3 cells, stably expressing NSD3 shRNA ("shNSD3-S5"), the scramble control shRNA ("shC"), lenti-CRISPR/Cas9 NSD3 sgRNA construct ("koNSD3") were established; Expression of *NSD3* mRNA was tested by qRT-PCR assays (**A**); Cells were further cultured for applied time periods, cell proliferation (nuclear EdU staining assays, **B**), migration ("Transwell" assays, **C**) and apoptosis (by recording nuclear TUNEL ratio, **D**) were tested. Data were presented as mean ± standard deviation (SD, n=5). ****P*** < 0.05 *vs.* "shC"/"koC" cells. Experiments in this figure were repeated five times with similar results obtained. Scale bar=100 μm (**B**).
